# From Alveolar Injury to Precision Perioperative Care: Integrating Molecular Biomarkers and Technology‐Enabled Strategies for Prolonged Air Leak After Lung Resection

**DOI:** 10.1155/mi/4062230

**Published:** 2026-08-04

**Authors:** Mahdi Ahmadinia, Seyed Hootan Hamidi, Behnaz Gholizadeh Niari, Amir Farrokhian, Ali Bozorg Savoji, Maryam Abbasi, Faezeh Jamali, Hamidreza Jamaati

**Affiliations:** ^1^ Lung Transplantation Research Center, National Research Institute of Tuberculosis and Lung Diseases (NRITLD), Shahid Beheshti University of Medical Sciences, Tehran, Iran, sbmu.ac.ir; ^2^ Department of Pharmacology and Toxicology, School of Pharmacy, Iran University of Medical Sciences, Tehran, Iran, iums.ac.ir; ^3^ Functional Neurosurgery Research Center, Shohada Tajrish Comprehensive Neurosurgical Center of Excellence, Shahid Beheshti University of Medical Sciences, Tehran, Iran, sbmu.ac.ir; ^4^ Department of Clinical Pharmacy, School of Pharmacy, Shahid Beheshti University of Medical Sciences, Tehran, Iran, sbmu.ac.ir; ^5^ Tehran Medical Branch, Islamic Azad University, Tehran, Iran, iautmu.ac.ir; ^6^ Department of Pulmonology, Chronic Respiratory Diseases Research Center, National Research Institute of Tuberculosis and Lung Diseases (NRITLD), Masih Daneshvari Hospital, Shahid Beheshti University of Medical Sciences, Tehran, Iran, sbmu.ac.ir; ^7^ Chronic Respiratory Disease Research Center, National Research Institute of Tuberculosis and Lung Diseases (NRITLD), Shahid Beheshti University of Medical Sciences, Tehran, Iran, sbmu.ac.ir

**Keywords:** biomarkers, lung resection, precision medicine, prolonged air leak, thoracic surgery

## Abstract

Prolonged air leak (PAL) remains one of the most frequent and clinically consequential complications after lung resection, prolonging chest tube duration, delaying recovery, increasing postoperative morbidity, and expanding healthcare resource utilization. Although traditionally approached as a mechanical failure of alveolar–pleural sealing or surgical technique, PAL is increasingly understood as a heterogeneous postoperative syndrome in which persistent air leak dynamics intersect with impaired inflammatory and reparative biology. Surgical manipulation, one‐lung ventilation, parenchymal injury, and patient‐specific vulnerability may activate cytokine signaling, epithelial barrier disruption, oxidative stress, extracellular matrix remodeling, glycocalyx degradation, and delayed wound repair. This narrative review synthesizes clinical, translational, and experimental evidence on the pathogenesis of PAL, with particular emphasis on inflammatory mediators and biomarker‐defined mechanisms relevant to failed closure of alveolar–pleural fistulas (APFs). Candidate biomarkers are discussed according to the biological domains they reflect, including systemic inflammatory burden and poor healing reserve (C‐reactive protein [CRP], IL‐6, and serum albumin), epithelial injury and damage‐associated signaling (high‐mobility group box 1 [HMGB1]/receptor for advanced glycation end products [RAGE] and sRAGE), oxidative epithelial stress (4‐hydroxynonenal [4‐HNE] and malondialdehyde [MDA]), protease‐mediated matrix and junctional remodeling (matrix metalloproteinase [MMP]‐9), glycocalyx and barrier disruption (syndecan [SDC]‐1), and upstream inflammatory regulation (histone deacetylase 6 [HDAC6]‐related pathways). The review also considers how quantitative digital chest drainage parameters may complement molecular biomarkers by capturing the physiologic expression of persistent air leak. By integrating these mechanistic and technological signals, this review proposes a conceptual framework for biomarker‐informed perioperative risk stratification, PAL phenotyping, and individualized prevention and management. Because most biomarker‐driven strategies remain investigational, prospective validation is required before routine clinical implementation.

## 1. Introduction

Prolonged air leak (PAL), conventionally defined as an air leak that persists beyond the fifth postoperative day (POD), remains one of the most frequent complications after lung resection and typically evolves from a postoperative air leak subset that fails to resolve within the expected early healing window [1]. PAL is clinically consequential rather than merely inconvenient: in a contemporary U.S. database analysis, patients with PAL had longer ICU stays, greater hospital costs, higher readmission risk, and nearly double the inpatient mortality of those without PAL [2]. Moreover, patients with poor healing reserve, including those with low albumin, impaired DLCO, and systemic steroid exposure, appear less likely to achieve spontaneous closure, suggesting that PAL reflects biological vulnerability as well as technical failure [3]. Yet, current management remains largely empirical and time‐based, and even recent trials using digital drainage systems show that chest tube strategy, suction level, and removal thresholds remain protocol‐dependent rather than biology‐informed [4].

This limitation matters because lung resection imposes a multihit injury on the alveolar surface through surgical manipulation, one‐lung ventilation, and postoperative inflammatory stress. In a nested case–control study after lung resection, IL‐6 rose systemically and was higher in the ventilated lung of patients who developed postoperative respiratory failure, supporting the relevance of compartmentalized cytokine signaling in perioperative lung injury [5]. At the same time, high‐mobility group box 1 (HMGB1) is a prototypical damage‐associated molecular pattern (DAMP) released during cellular stress and oxidative injury that signals through receptor for advanced glycation end products (RAGE) and toll‐like receptor (TLR)2/4/9 to amplify nuclear factor kappa‐B (NF‐κB) activation and downstream cytokine production [6]. This is mechanistically relevant because RAGE is highly expressed in alveolar epithelial cells (AECs), sRAGE correlates with IL‐6 and IL‐8 in experimental lung injury, and RAGE inhibition attenuates epithelial apoptosis and barrier dysfunction [7]. Cytokine networks may also influence failed repair through remodeling pathways, as tumor necrosis factor (TNF)‐superfamily signaling has been implicated in epithelial–fibroblast crosstalk, tissue remodeling, and fibrosis across inflammatory lung contexts [8].

Downstream barrier failure provides a plausible bridge from inflammation to persistent alveolar–pleural fistula (APF) closure failure. In experimental lung injury, increased matrix metalloproteinase [MMP]‐9 activity and a higher MMP‐9/tissue inhibitor of metalloproteinases (TIMP)‐1 ratio reduce E‐cadherin and occludin, thereby weakening epithelial junctional integrity [9]. A closely related work shows that MMP‐9‐mediated syndecan [SDC]‐1 shedding aggravates alveolar tight‐junction injury and permeability, linking glycocalyx disruption to delayed barrier recovery [10]. More broadly, circulating SDC‐1 is an established marker of glycocalyx injury in clinical medicine and may therefore serve as a translational indicator of perioperative barrier damage [11]. Oxidative stress adds another layer: 4‐hydroxynonenal (4‐HNE) and malondialdehyde (MDA) are reliable lipid peroxidation products associated with alveolar epithelial injury in contemporary lung‐disease models, supporting their candidacy as markers of oxidative epithelial stress rather than as PAL‐specific predictors at present [12]. Among more accessible biomarkers, hypoalbuminemia in chronic lung disease reflects impaired nutritional, anti‐inflammatory, and antioxidant reserve, whereas C‐reactive protein (CRP) captures the systemic inflammatory burden but remains biologically nonspecific [13]. Upstream regulatory pathways such as histone deacetylase 6 (HDAC6) are also increasingly relevant because selective HDAC6 inhibition has been shown to reduce IL‐6 and TNF‐α while preserving ZO‐1 and E‐cadherin in injured airway epithelium [14].

This narrative review therefore examines the pathogenesis of PAL through an inflammatory mediator–centered framework, positioning persistent postoperative air leak as a heterogeneous syndrome arising from the interaction of mechanical air‐leak dynamics with alveolar epithelial injury, cytokine‐driven inflammation, oxidative stress, extracellular matrix remodeling, glycocalyx disruption, and impaired wound repair. We synthesize current clinical, translational, and experimental evidence on candidate biomarkers that reflect these interconnected domains and explore how they may be integrated with conventional clinical risk factors and digital air‐leak measurements to support a more precise perioperative framework for PAL risk stratification, phenotyping, prevention, and management.

## 2. Methods

This comprehensive narrative review was conducted to synthesize contemporary evidence on the pathophysiology, biomarkers, and emerging management strategies for PAL following lung resection. The literature search focused on the peer‐reviewed articles published between January 2015 and December 2025. Four major biomedical databases were selected a priori as primary sources of evidence: PubMed/MEDLINE, Scopus, Web of Science, and Embase. These databases were chosen to ensure comprehensive coverage of clinical, translational, and experimental studies relevant to thoracic surgery, pulmonary medicine, perioperative care, and biomarker research.

Searches were performed using combinations of keywords and medical subject headings related to “prolonged air leak,” “postoperative air leak,” “lung resection,” “thoracic surgery,” “biomarkers,” “digital chest drainage,” “pleurodesis,” and “pharmacologic therapy.” Reference lists of key articles were also manually screened to identify additional relevant publications.

Eligible studies included original research articles, randomized controlled trials (RCTs), observational cohort studies, systematic reviews, and meta‐analyses published in international journals. Preclinical and translational studies were considered when they provided mechanistic insights directly relevant to epithelial injury, impaired lung repair, inflammatory activation, extracellular matrix remodeling, or potential therapeutic targets in the context of PAL biology. Editorials, isolated case reports, conference abstracts, non–peer‐reviewed sources, and articles without clear relevance to PAL pathophysiology, biomarker interpretation, or management were excluded from the narrative synthesis.

Article selection was performed qualitatively based on topical relevance, clinical or mechanistic importance, and contribution to the conceptual framework of biomarker‐guided PAL management. The evidence was synthesized thematically rather than quantitatively, with emphasis on distinguishing PAL‐specific clinical evidence from broader perioperative, indirect lung‐injury, translational, and preclinical evidence. Given the narrative design of this review, no PRISMA‐based screening flow diagram, formal risk‐of‐bias assessment, quality scoring, or quantitative meta‐analysis was undertaken. Accordingly, the resulting synthesis is intended to provide a structured conceptual framework and research‐oriented perspective rather than prescriptive clinical recommendations.

## 3. Pathophysiology of PAL in Thoracic Surgery

### 3.1. Local and Anatomical Mechanisms

PAL results from a failure of the normal lung sealing mechanisms, typically due to an APF—a persistent communication between the lung parenchyma and pleural space [15]. Fundamental mechanisms include extensive parenchymal injury (including surgical or emphysematous lung damage) and impaired visceral pleural healing. A large initial lung injury or incomplete fissure requiring extensive dissection can create a sizeable air leak, and delayed apposition or scarring of the pleural surfaces prevents timely closure [16]. For instance, PAL is often precipitated by an intraoperative tear or a staple‐line defect that fails to seal because the remaining lung cannot fully re‐expand to contact the chest wall, leaving a residual pleural space [17]. In patients with advanced pulmonary emphysema or poor lung quality, the friable parenchyma is prone to such fistula formation. Indeed, chronic obstructive pulmonary disease (COPD) is one of the most consistent risk factors for PAL [18, 19]. Patients with reduced preoperative lung function (like FEV_1_ <70% predicted) have a markedly higher incidence of postoperative air leaks [20]. In a recent video‐assisted thoracoscopic surgery (VATS) series, the presence of COPD increased the odds of PAL by approximately 9‐fold (OR ≈9.0) [21]. Additionally, extensive pleural adhesions from prior inflammation (including old tuberculosis or empyema) can contribute—adhesiolysis causes lung surface tears and these patients show high PAL rates [19, 21]. For example, resection in multidrug‐resistant tuberculosis yielded a 33.3% PAL incidence in one report [19], underscoring how chronic inflammation and scarring hamper pleural healing.

### 3.2. Systemic and Nutritional Factors

Systemic factors play a pivotal role in modulating tissue repair capacity. Malnutrition and hypoalbuminemia are strongly linked to PAL. A retrospective study of 146 lobectomy patients identified low preoperative serum albumin as an independent predictor of PAL [19, 22]. The authors noted that hypoalbuminemia reflects poor nutrition, which impairs wound healing and collagen synthesis in lung tissues [23, 24]. Sarcopenia (loss of skeletal muscle mass) similarly indicates frailty and suboptimal reserves for healing. In patients with early‐stage lung cancer, those in the lowest quartile of muscle mass (psoas volume index) had a PAL frequency of 16.9%, nearly double that of patients with normal muscle metrics (9.6%) [25]. Low muscle mass was associated with over twice the odds of major complications post‐lobectomy [25]. Even outside the surgical setting, poor nutritional status predisposes individuals to persistent air leaks. In spontaneous pneumothorax, a low geriatric nutritional risk index conferred a hazard ratio greater than 2.5 for continued air leak beyond 7 days [26].

### 3.3. Systemic Inflammatory and Immunologic Factors

A pro‐inflammatory systemic milieu is another detrimental factor. Elevated inflammatory biomarkers like CRP and cytokines signal a heightened immune response that may impede tissue repair. High postoperative IL‐6 and CRP levels have been associated with increased risk of complications such as infections [27], suggesting that an exaggerated inflammatory state correlates with poorer healing. While specific data linking IL‐6 or TNF‐α directly to PAL risk are still emerging, experimental evidence shows that TNF‐α can disrupt normal wound‐healing pathways by inhibiting collagen deposition [8]. Chronic inflammatory lung diseases exemplify this effect: patients with ongoing inflammation (such as active tuberculosis and aspergillosis) suffer higher PAL rates after resection [19]. The tissue inflammation and fibrosis in these conditions likely compromise the lung’s ability to seal air leaks. Furthermore, chronic corticosteroid use—often prescribed for COPD or other inflammatory conditions—markedly increases the PAL risk [15, 20]. Steroids blunt the inflammatory response but also impair fibroblast function and wound healing in the pleura [28]. Patients on >10 mg prednisone (or equivalent) for >1 month preoperatively are considered high‐risk for PAL [19]. Indeed, one analysis identified preoperative steroid use as a significant predictor of PAL alongside smoking and low FEV_1_ [20].

### 3.4. Physiologic Air Leak Dynamics

Beyond patient factors, the physiologic characteristics of the air leak itself are critical to PAL pathophysiology. Early postoperative air leak metrics—now measurable with digital chest drainage systems—can predict which leaks will persist. Airflow magnitude and pattern in the first 24–48 h are key indicators. Larger initial air leaks (high air flow rates) are much more likely to be prolonged [29]. For example, one study found that an air leak >50 mL/min measured at 6 h post‐lobectomy, especially when combined with large intrapleural pressure swings (>10 cmH_2_O), carried over a 50% risk of evolving into PAL [30]. By contrast, small early leaks often seal spontaneously within a few days [30]. The nature of the leak pattern (continuous vs. intermittent) also matters. Using a digital drainage device, Shintani et al. [31] categorized post‐lung resection leaks into four patterns: none, intermittent, decreasing, and variable. Strikingly, patients with a “variable” air leak pattern (fluctuating and high‐output leak) had a 76% incidence of PAL, whereas those with no air leak or only minimal intermittent leaks on day 1 rarely developed PAL (≤1% incidence) [31]. An intermediate pattern (gradually decreasing leak) had about a 20% PAL rate [19]. Thus, a persistently high or erratic air leak in the immediate postoperative period is a red flag for prolonged leakage. Clinically, even with traditional chest drains, the presence of a substantial air leak on POD one (including air bubbling at rest) is known to predict PAL [32]. Modern digital chest drainage systems enhance this assessment by providing precise, continuous measurements of air leak flow and intrathoracic pressure. These systems not only objectify the air leak size (in mL/min) but also record pleural pressure trends, aiding in early risk stratification [31, 33–35]. Studies confirm that pleural pressure readings can help identify patients at risk for PAL [33, 35]. For instance, a higher pleural pressure differential (Δ*P*) during respiration indicates greater air leak flow under pressure and correlates with prolonged leaks [30]. Real‐time digital monitoring has shown that patients with large pleural pressure swings (Δ*p*  > 10 cmH_2_O) and high leak flow soon after surgery have up to a 52% probability of PAL if no intervention is done [30]. In contrast, low initial flows and pressure swings carry minimal risk (as low as ~4%) [30, 34]. Moreover, digital devices allow the detection of air leak patterns (e.g., continuous vs. only with cough), which further informs prognosis [31].

### 3.5. Lung Mechanics and Postoperative Expansion in the Interpretation of Air Leak

Postoperative air leak after lung resection should not be interpreted solely as a surrogate of fistula size or measured flow because the persistence of air loss also reflects how the remaining lung re‐expands within the thoracic cavity, how pleural pressure behaves during respiration, and whether pleural apposition is restored after closure [36–38]. Residual pleural space is shaped by parenchymal compliance, elastic recoil, diaphragmatic and mediastinal shift, and the absorptive capacity of the pleural compartment; when expansion is incomplete, mechanical separation of visceral and parietal pleura can delay spontaneous sealing of an APF [36, 38, 39]. This mechanism is especially relevant in emphysema, reduced recoil, upper lobectomy, incomplete fissures, pleural adhesions, and larger resections, all of which are associated with PAL risk or persistent postoperative pleural space [20, 40]. Digital drainage studies further show that quantitative airflow trajectories and pleural pressure fluctuations provide clinically useful information beyond binary bubbling because worsening or highly variable leak patterns and delayed recovery of pleural pressure swings are linked to PAL persistence [35, 41]. Accordingly, drainage strategy should be individualized according to the combined context of air‐leak magnitude, radiographic lung expansion, residual pleural space, pleural pressure data, and overall clinical stability rather than flow alone [42, 43]. Suction may be helpful when lung expansion is incomplete or a residual space is clinically relevant, but routine or excessive suction does not uniformly accelerate closure, and, in selected cohorts, water seal or nonsuction strategies have yielded shorter drainage times and fewer pleural interventions or less subcutaneous emphysema [4, 44, 45]. In this framework, a mechanical‐dominant PAL phenotype can be recognized by persistent leak accompanied by incomplete expansion, residual pleural space, marked pressure swing abnormalities, or worsening subcutaneous emphysema, and such patients may warrant closer monitoring, drainage adjustment, repeat imaging, or earlier pleurodesis, bronchoscopic, or surgical evaluation [1, 3, 4].

## 4. Biomarkers Relevant to PAL

Current evidence regarding the precise cellular and molecular mechanisms underlying PAL after lung resection, as well as the specific biomarkers that predict air leak duration before and after surgery, remains limited. However, based on the pathophysiology of air leak, factors that disrupt epithelial cell integrity in the lung and increase inflammation are likely to impair cellular and tissue repair, thereby contributing to the development of PAL. The following section introduces potential biomarkers that may be relevant. Investigating and monitoring these factors could be essential for developing clinical algorithms to predict PAL following lung resection.

### 4.1. HMGB1 Protein

HMGB1 protein is a nuclear DNA‐binding protein that maintains the structure and stability of chromosomes. It can also be secreted extracellularly as a DAMP molecule from damaged cells or various activated cells. HMGB1 is known as an inflammatory agent in many pathological conditions of the lungs. It contributes to lung inflammation through multiple mechanisms, mostly by interacting with receptors such as RAGE, TLR2, TLR4, and TLR9, which activate proinflammatory signaling pathways [6, 46, 47].

Many studies show HMGB1 is a potential candidate for worsening the inflammation of lung disease and its prognostic biomarker for predicting the incidence such as asthma, COPD, pulmonary fibrosis, and acute respiratory distress syndrome (ARDS) [6, 46–49].

In several studies, HMGB1 enhances the permeability of the airway epithelial barrier in a manner dependent on both concentration and exposure time. HMGB1 downregulates the levels of occludin and claudin‐1, ‐4, and ‐7, induces the redistribution of E‐cadherin and β‐catenin, and increases claudin‐2. Thus, HMGB1 compromises epithelial barrier integrity by perturbing the structural organization of tight and adherens junctions (AJs), ultimately resulting in increased paracellular permeability and epithelial dysfunction [50–53].

### 4.2. RAGE

One of the important ways that HMGB1 induces disruption of epithelial integrity and inflammation is the activation of the RAGE, which is a transmembrane pattern recognition receptor that is expressed in mammals and belongs to the immunoglobulin (Ig) superfamily [7, 54, 55]. It is highly expressed in AECs and is a biomarker of epithelial injury [7, 56].

Calfee et al. [57] found that plasma RAGE concentration observed in patients ventilated with higher tidal volumes is higher than those ventilated with lower tidal volumes, and it may be used in monitoring ventilator‐induced injury. In addition, a study by Zhai et al. [58] demonstrated that RAGE by activation of RhoA, increased permeability, inflammation, lung injuries and impaired oxygenation in both in vivo and vitro models of sterile lung injury. sRAGE is the main soluble form of RAGE and shows the strongest potential as a biomarker for lung epithelial injury in clinical practice. In one meta‐analysis, patients with higher baseline plasma sRAGE have higher degrees of lung epithelial injury [59]. Therefore, elevated concentrations of sRAGE in the bloodstream suggest that the cell surface receptor RAGE has been excessively stimulated, a condition that, if sustained, could amplify proinflammatory mechanisms and worsen pathological conditions [7].

### 4.3. CRP

CRP is a liver‐derived acute‐phase protein that rises in response to systemic inflammation. It is not disease‐specific; CRP provides an insight into the overall inflammatory state of the body. Research across multiple respiratory conditions has shown that the level of CRP in plasma can be a prognostic and predictive biomarker, impaired functional status, metabolic alterations, and some cases, responsiveness to therapy. Its utility has been demonstrated in COPD, interstitial lung diseases, and non‐small cell lung cancer, indicating that CRP measurement can offer valuable information regarding disease severity and progression in a clinical setting [54, 60–62]. However, in the case of PAL, data about the role of CRP as a biomarker is limited and was contradictory and to some extent dismissive of it.

In a retrospective study of patients undergoing VATS, CRP levels were compared between those who developed PAL and those who did not. No significant difference was observed between the groups, suggesting that systemic inflammation, as reflected by CRP, may not directly contribute to the occurrence or persistence of postoperative air leaks [63]. However, a study by Lopez‐Pastorini et al. [64] indicated that in patients with impaired anastomotic healing, postoperative complications such as PAL are more frequent. Additionally, elevated preoperative CRP is associated with a higher risk of anastomotic healing impairment and, consequently, increased likelihood of air leak [65]. In another investigation, patients with COVID‐19 who required high‐flow nasal cannula (HFNC) support developed higher CRP levels. However, when controlling for other variables, CRP did not emerge as an independent predictor of AL. CRP may reflect more severe lung inflammation and a generally worse clinical condition, rather than directly causing air leak. On the other hand, mechanical factors, such as increased transpulmonary driving pressure, positive end‐expiratory pressure, and pressure support ventilation, along with higher baseline D‐dimer and consolidative patterns on CT scans, were found to be directly associated with the onset of AL. Thus, while HFNC patients with high CRP appear more vulnerable, the main contributors to air leak seem to be mechanical stress and underlying lung injury, with CRP serving primarily as a marker of disease severity [66].

### 4.4. Serum Albumin

Serum albumin, as a biomarker, serves not only to indicate the nutritional condition of the body but also to eliminate pro‐inflammatory stimuli and mitigate inflammatory responses, thereby reflecting the degree of systemic inflammatory status to some extent [65].

Hypoalbuminemia has often been viewed as an indicator of inadequate nutritional status; however, plasma albumin levels are affected by several factors in addition to hepatic synthesis, such as shifts in plasma volume and redistribution among vascular compartments. As a result, albumin concentration is not a dependable marker in numerous clinical scenarios, including inflammation, metabolic disorders, liver disease, and surgical tissue trauma [66].

Various research focuses on albumin concentration levels in types of lung diseases, specifically in lung cancers. In most of them, albumin is a meaningful biomarker when it comes to the other parameters [13, 65, 67, 68]. In the context of PAL, the same thing was observed. In a study on 146 patients, the preoperative serum albumin level and the presence of a visually evaluated air leak on POD 1 were independent predictors of PAL [23]. A reduction in serum albumin levels, established at a cut of 14.97%, could serve as an indicator for identifying patients who are at considerable risk of experiencing postoperative pulmonary complications, including AL, following thoracoscopic anatomical lung cancer surgery [69]. Dezube et al. [3] discovered that in a subanalysis of PAL patients necessitating intervention, poorer FEV1%, diminished DLCO%, reduced albumin levels, and heightened steroid usage are associated with a greater likelihood of requiring intervention. However, in another study on 493 patients after VATS pulmonary resection, data demonstrated that postoperative serum albumin was significantly lower in patients with PAL in univariate analysis; this variable did not remain an independent predictor in multivariate logistic regression. This suggests that low albumin may reflect a general poor condition rather than a direct causal factor for PAL [70].

### 4.5. 4‐HNE

During oxidative stress, cells experience lipid peroxidation reactions that yield harmful oxidized by‐products, including 4‐HNE and MDA. 4‐HNE is an endogenous α, β‐unsaturated hydroxyalkenal produced at concentrations ranging from 0.1 to 3 μM under normal physiological conditions. However, in conditions of oxidative stress, the concentration of 4‐HNE that accumulates in cellular membranes can vary from 10 to 5 mM. In response to hyperoxia‐induced oxidative stress, physiological levels of 4‐HNE are recorded to be below 20 ng/mg of protein. Furthermore, 4‐HNE can cause protein dysfunction by forming adducts with lysine, histidine, and cysteine residues, in addition to generating stable Michael addition products and disulfide bonds. These harmful reactions ultimately contribute to 4‐HNE‐induced cellular apoptosis and cell death [71].

The increased concentrations of 4‐HNE could be involved in the signaling processes associated with lung inflammation, resulting in an imbalance in the expression of proinflammatory mediators and protective antioxidant genes in COPD [72]. HNE triggered both apoptosis and ferroptosis in mouse lung epithelial cells [73].

### 4.6. MMP 9

MMPs are a family of proteolytic enzymes, including gelatinases, collagenases, stromelysins, matrilysins, and membrane‐type MMPs. MMP‐9 belongs to one of the MMPs and has a critical role in the pathogenesis of inflammatory diseases with systemic or local destruction, such as sepsis, rheumatoid arthritis, and lung injury. MMP‐9 can be secreted by many cell types, including neutrophils, macrophages, lung epithelial cells, and fibroblasts [74]. The main function of MMP‐9 is to damage and reshape the dynamic balance of the extracellular matrix. MMP‐9 plays an important role in the degradation and remodeling of the alveolar–capillary membrane and maintenance of the integrity of the basement membrane. A study showed that the high ratio of MMP‐9/TIMP‐1 decreased E‐cadherin and occludin in ventilator‐induced lung injury (VILI) in vivo. MMP‐9 knockdown decreased the ratio of MMP‐9/TIMP‐1 and reversed the expressions of E‐cadherin and occludin to alleviate the lung structure in VILI [9].

Plasma MMP‐9 level increased after VATS lobectomy. Paravertebral block (PVB) inhibited surgical stress and decreased postoperative MMP‐9 level, attenuation of MMP‐9 response to surgery, and provided statistically better pain relief after VATS lobectomy. This technique may be beneficial for patients to recover rapidly after lung surgery and reduce tumor recurrence [75]. Elevated MMP‐9 is associated with accelerated lung function decline and COPD development [76]. MMP9 exerts its effects on the epithelium by cleaving one or more components of cell–cell junctions and triggering anoikis. Taken together, these data suggest that a component of airway remodeling associated with asthma may be directly regulated by MMP9 [77]. Following VATS lobectomy, there was an increase in plasma MMP‐9 levels. PVB was effective in reducing surgical stress and lowering postoperative MMP‐9 levels, which in turn diminished the MMP‐9 response to surgery and provided statistically improved pain relief after VATS lobectomy. This method could be beneficial for patients, promoting rapid recovery after lung surgery and decreasing the likelihood of tumor recurrence [9].

### 4.7. Glycocalyx and SDC‐1

The glycocalyx constitutes a dynamic extracellular layer composed of glycoproteins and proteoglycans that plays a central role in regulating vascular permeability, inflammatory responses, shear stress, and fluid homeostasis. This barrier is supplemented by intercellular junctions (especially tight junctions [TJs] in the pulmonary microvasculature) and regulates parenchymal permeability. Inflammatory stimuli under tissue injury conditions activate MMPs and other proteases that degrade elements of heparan sulfate proteoglycans (HSPG) and chondroitin sulfate proteoglycans (CSPG) components and, as a result, cause shedding of epithelial and endothelial glycocalyx (EGL). This process increases the alveolar permeability, breaks the functions of surfactants, increases the virulence of bacteria, and diminishes the repair of epithelial cells [10, 78–80].

SDC‐1 is a major HSPG and a core structural component of the glycocalyx on both endothelial and epithelial cell surfaces. In situations that cause glycocalyx damage, including inflammation, surgical stress, or mechanical trauma, SDC‐1 is released into the bloodstream, rendering it a sensitive biomarker of EGL injury in clinical diagnosis. SDC‐1 is also expressed in AECs in the lung, where it facilitates epithelial adhesion, migration and repair. Thus, the shedding of SDC‐1 not only indicates the disruption of the endothelial barrier but could also play a role in impaired epithelial regeneration, elevated permeability, and increased susceptibility to lung injury [10, 11, 78–80].

VATS was linked to a major loss of the EGL, as indicated by a drastic increase in the levels of SDC‐1. SDC‐1 went up by almost 20% during the immediate postoperative period in patients who did not get defensive measures and by greater amounts in surgeries lasting longer than 3 h, highlighting a direct duration‐dependent interference of the glycocalyx itself. Conversely, the levels of heparin sulfate did not change significantly, which can be explained by quick clearance by the kidneys. The observed decrease in plasma albumin that was more than that in hemoglobin also supports the hypothesis of increased microvascular permeability by glycocalyx degradation. These data indicate the critical importance of glycocalyx in maintaining vascular barrier integrity in pulmonary surgery and indicate that even minor surgery like VATS can cause measurable damage to the endothelial surface layer [78].

In the study, the authors demonstrated that protecting SDC‐1 shedding significantly preserves alveolar epithelial TJ integrity and reduces LPS‐induced lung injury. The findings also highlight close interactions between SDC‐1, TJs, AJs, and gap junction proteins such as connexin 43 in early acute lung injury (ALI) [10]. In the other study, deletion of SDC‐1 was shown to exacerbate lung injury in influenza infection, as SDC‐1 normally prevents epithelial apoptosis and widespread inflammation by activating the protective c‐Met pathway [81].

### 4.8. HDAC 6

HDAC6 is highly cytoplasmic and is a type of deacetylase that controls the most important non‐histone substrates, such as 2‐tubulin, as well as other signaling proteins, thus affecting cytoskeleton dynamics, ciliary activity, and response to stress in lung epithelial cells. In pulmonary disease, HDAC6 activity has been linked to augmented epithelial inflammation, facilitating epithelial–mesenchymal transition (EMT), and destabilizing epithelial integrity; and selective silencing of HDAC6 has been evidenced to protect epithelial organization, cytokine system, and fibrotic remodeling in experimental models [82, 83].

It was shown that HDAC6 inhibition with ACY‐1083 protects airway epithelial cells from COPD‐relevant challenges, preserving barrier integrity, mucociliary function, and reducing cytokine and mucin production. These effects occurred without directly altering the ciliary beat frequency or TJ proteins, suggesting alternative protective mechanisms. HDAC6 inhibition may thus restore epithelial structure and function and help halt COPD progression [83]. A second piece of literature indicated that exposure to cigarette smoke increases the expression of HDAC6 in the murine lung tissue and in human bronchial epithelial cells. Selective HDAC6 inhibitor CAY10603 was used to reduce emphysematous alterations, recover the expression of epithelial junctional proteins, and inhibit transforming growth factor‐1 (TGF‐1)‐mediated Smad 2 /3 signaling and EMT, decreasing remodeling of the small airways [14]. A study noted that LPS exposure increased the activity of HDAC6 in lung tissue, which was accompanied by the reduction of α‐tubulin acetylation and impaired epithelial integrity. Selective HDAC6 inhibitor CAY10603 treatment averted deacetylation of alpha‐tubulin, decreased pro‐inflammatory cytokine synthesis and infiltration of leukocytes, maintained E‐cadherin expression, and suppressed the expression of MMP9. NF‐κB activation and inflammasome signaling inhibition were also prevented by HDAC6 inhibition and had a protective effect on acute inflammatory lung injury [84]. In another study, it was shown that nickel nanoparticles (Nano‐Ni) induce pulmonary inflammation and fibrosis by activating the NLRP3 inflammasome. This activation depends on HDAC6 upregulation and acts as a secondary signal in LPS‐primed alveolar macrophages, leading to IL‐1β production. Importantly, Nano‐Ni caused only mild pulmonary damage in Nlrp3^–/–^ or Il‐1r1^–/–^ mice, highlighting the critical role of HDAC6‐mediated NLRP3 activation in Nano‐Ni‐induced lung injury [85].

**Figure 1:**
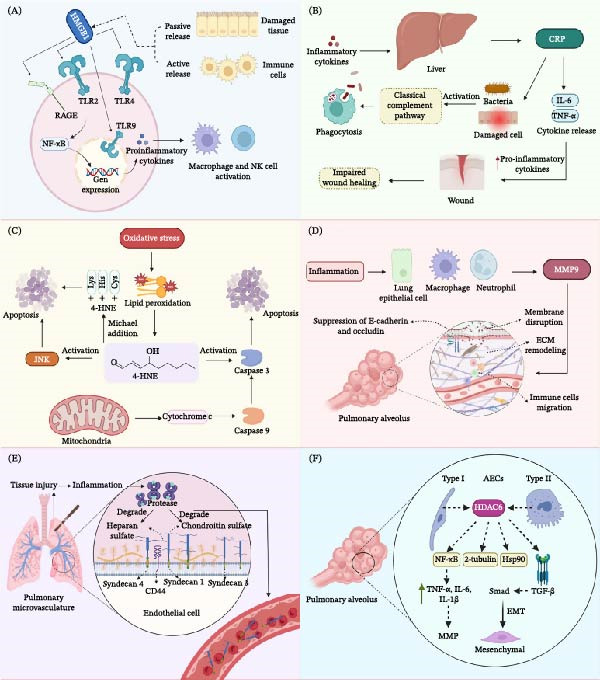
Schematic representation of biomarker‐related molecular mechanisms contributing to prolonged air leak (PAL). (A–F) It illustrates the signaling pathways mediated by HMGB1/RAGE, CRP, 4‐HNE, MMP‐9, glycocalyx, and HDAC6. (A) HMGB1/RAGE axis: HMGB1 is released actively from immune cells or passively from necrotic epithelial cells and binds to RAGE, TLR2, TLR4, and TLR9, triggering the NF‐κB signaling cascade. Activated NF‐κB translocates into the nucleus and induces transcription of pro‐inflammatory cytokines (IL‐6 and TNF‐α), leading to macrophage and NK cell activation. (B) C‐reactive protein (CRP): CRP is secreted by hepatocytes during inflammation. It binds to damaged cells and bacterial membranes, promoting opsonization and classical complement pathway activation. In addition, CRP amplifies pro‐inflammatory cytokine production and delays epithelial repair processes. (C) 4‐Hydroxynonenal (4‐HNE): lipid peroxidation under oxidative stress generates 4‐HNE; the apoptotic pathways induced by 4‐HNE include activation of caspase‐3/caspase‐9 and cytochrome c release from mitochondria and JNK. In addition, it forms adducts with lysine, histidine, and cysteine residues, which leads to apoptosis. (D) Matrix metalloproteinase‐9 (MMP‐9): inflammatory stimuli induce MMP‐9 release from macrophages, neutrophils, and alveolar epithelial cells. MMP‐9 degrades ECM components and disrupts tight‐ and adherens‐junction proteins (E‐cadherin and occludin) and alveolar membrane, increasing alveolar permeability and promoting persistent air leakage. (E) Glycocalyx: glycocalyx and syndecan‐1 in pulmonary and blood vessel cells structure components are CD44, syndecan‐1, 3, and 4, and they are involved in regulating vascular permeability. Tissue injury triggered inflammation factors and caused the activation of MMPs. MMPs also caused degradation of syndecan 1 and its release to the blood vessel. (F) HDAC6: AECs are of two types, type 1 and type 2, which become mesenchymal during the EMT process. Their cytoplasm contains HDAC6, which plays a role in the deacetylation of tubulin and Hsp90, also regulates the NF‐κB mechanism and ultimately increases inflammatory cytokines. In addition, HDAC6 helps the EMT process by affecting the TGF‐β and Smad mechanisms. 4‐HNE, 4‐hydroxynonenal; AECs, alveolar epithelial cells; CRP, C‐reactive protein; ECM, extracellular matrix; EMT, epithelial–mesenchymal transition; HDAC6, histone deacetylases 6; HMGB1, high‐mobility group box 1 protein; JNK, c‐Jun N‐terminal kinases; MMP, matrix metalloproteinase; NF‐κB, nuclear factor kappa‐B; PAL, prolonged air leak; RAGE, receptor for advanced glycation end products; TGF‐β, transforming growth factor‐beta; TLR, toll‐like receptor.

An overview of candidate biomarkers implicated in the prediction and pathophysiology of PAL following lung resection is provided in Table 1. Figure 1 shows a schematic illustration of signaling pathways that HMGB1/RAGE, CRP, 4‐HNE, and MMP‐9 contribute to epithelial disruption, oxidative stress, inflammation, and delayed wound repair, which could lead to PAL.

**Table 1:** Candidate biomarkers relevant to the prediction and pathophysiology of PAL after lung resection.

Biomarker	Biological source	Principal mechanistic role	Pathophysiologic domain	Clinical relevance in PAL	Timing of assessment	References
Serum albumin	Serum (preoperative)	Maintains plasma oncotic pressure; supports tissue repair (nutritional status indicator)	(NA) Systemic nutritional/wound healing	Low preoperative albumin predicts higher PAL risk (poor healing)	Preoperative (baseline)	[3, 23]
Interleukin‐6 (IL‐6)	Serum (postoperative)	Pro‐inflammatory cytokine driving the acute phase response	Inflammation	Elevated IL‐6 post‐surgery correlates with complications (persistent inflammation); high early IL‐6 distinguishes high‐risk patients	Early postoperative (24–72 h)	[5, 86]
Surfactant protein D (SP‐D)	Lung epithelium (type II cells; circulating in serum)	Enhances innate immunity and surfactant homeostasis; a marker of alveolar epithelial integrity	Lung epithelial integrity	Lung injury causes SP‐D leakage into blood; elevated circulating SP‐D indicates alveolar damage and is significantly higher in ARDS patients (reflects severity of alveolar injury)	Preoperative or early postoperative (serum)	[87, 88]
Soluble RAGE (sRAGE)	Plasma (endothelial/epithelial junctions)	The shed receptor from alveolar type I cells reflects alveolo‐capillary barrier injury	Vascular–epithelial permeability	Validated prognostic biomarker of ARDS: elevated sRAGE indicates severe epithelial injury; strongly associated with ARDS development in at‐risk patients (impaired barrier function)	Immediate postoperative (plasma)	[89, 90]
Malondialdehyde (MDA)	Serum or exhalate (oxidative stress byproduct)	Lipid peroxidation end‐product indicating ROS‐induced cell membrane damage	Oxidative stress	High postoperative MDA signifies excessive oxidative injury and correlates with worse postoperative outcomes; unchecked oxidative stress leads to greater tissue damage and complications	Postoperative (first 7 days)	[91, 92]
Matrix metalloproteinase‐9 (MMP‐9)	Lung parenchyma (alveolar macrophages; also, in serum)	Proteolytic enzyme degrading extracellular matrix (collagen, elastin); mediates tissue remodeling	ECM remodeling	Overexpressed in diseased lung tissue; high MMP‐9 in alveolar macrophages correlates with alveolar wall destruction and recurrent air leaks. Increased MMP‐9 is linked to inflammatory lung injury and adverse outcomes	Intraoperative (resected lung tissue analysis); or early postoperative (serum)	[93, 94]
Angiopoietin‐2 (Ang‐2)	Plasma (endothelial cells)	Antagonist of Ang‐1/Tie2 signaling; increases vascular permeability and endothelial instability	Vascular–epithelial permeability	Elevated Ang‐2 levels indicate a compromised pulmonary microvascular barrier; they predict acute lung injury/ARDS onset in critically ill patients (associated with persistent air leak due to delayed barrier recovery)	Perioperative (pre‐ and early postoperative plasma)	[95, 96]
α1‐Antitrypsin (AAT)	Serum (preoperative)	Major inhibitor of neutrophil elastase; protects alveolar connective tissue from proteolytic degradation	Lung epithelial integrity/ECM	AAT deficiency leads to unopposed elastase activity, causing premature panacinar emphysema and subpleural bleb formation. Spontaneous pneumothorax (persistent air leak) can be a presenting complication of severe AAT deficiency	Preoperative (screening serum level)	[97]

## 5. Comparative Interpretation and Clinical Prioritization of Candidate Biomarkers

Candidate biomarkers implicated in PAL should not be regarded as equally clinically ready. At present, preoperative serum albumin and early quantitative air‐leak assessment are the most clinically actionable signals because hypoalbuminemia has been linked to PAL after lobectomy, perioperative albumin decline reflects impaired postoperative recovery, and digital drainage phenotypes can identify patients at risk for persistence and earlier intervention after lung resection [1, 23, 31, 69]. By contrast, CRP and IL‐6 are better interpreted as nonspecific markers of perioperative inflammatory burden; in thoracic surgery, they correlate more consistently with overall postoperative morbidity or infection than with PAL‐specific biology, which limits their value as stand‐alone decision tools for PAL management [27, 64].

Mechanistically, however, HMGB1/RAGE‐related epithelial injury provides an important translational framework. HMGB1 disrupts epithelial junctions and increases permeability, while RAGE signaling amplifies NF‐κB‐dependent inflammatory responses; circulating sRAGE, in turn, has shown value as a marker of alveolar epithelial injury in ARDS‐oriented studies rather than in PAL‐specific cohorts [46, 59, 98–100]. Downstream, inflammatory activation may enhance MMP‐9 signaling, promoting extracellular‐matrix degradation and loss of junctional proteins. In contrast, oxidative stress products, such as 4‐HNE and MDA, indicate lipid peroxidation and epithelial stress that may further delay repair [9, 12]. Glycocalyx injury, reflected by SDC‐1 shedding, may compound barrier dysfunction, and lung resection itself has been associated with measurable glycocalyx disruption [10, 101]. HDAC6 remains even further from routine use, serving mainly as an upstream mechanistic regulator of inflammatory signaling, epithelial barrier instability, and remodeling in preclinical lung‐injury models [14, 102]. PAL is, therefore, better conceptualized as a heterogeneous syndrome spanning mechanical, inflammatory, epithelial‐injury, extracellular matrix‐remodeling, and poor‐healing phenotypes. Because validated PAL‐specific thresholds, standardized sampling windows, and prospective validation are still lacking, the near‐term clinical utility of these markers will most likely come from multiparametric panels integrated with clinical risk factors and digital drainage data rather than from any single biomarker in isolation [1, 31, 59, 100]. A comparative prioritization of candidate biomarkers according to their PAL‐specific evidence, clinical readiness, potential role in patient management, and major limitations is presented in Table 2.

**Table 2:** Comparative prioritization of candidate PAL biomarkers.

Biomarker	Evidence in PAL	Clinical readiness	Potential role	Main limitation	References
Serum albumin	The strongest direct PAL‐specific clinical signal among circulating biomarkers remains preoperative hypoalbuminemia. In a lobectomy cohort, lower albumin independently predicted PAL, suggesting a poor‐healing or impaired‐reserve phenotype rather than a lung‐specific injury pathway	Moderate	Preoperative risk enrichment: identify patients who may benefit from nutritional optimization and closer postoperative monitoring	Nonspecific; affected by nutrition, liver function, inflammation, and perioperative fluid shifts; limited PAL‐specific validation	[23]
Digital air‐leak parameters	This is the most actionable postoperative “marker‐like” signal in current practice. Digital drainage studies show that objective flow measurements and early leak patterns predict PAL, while broader implementation studies suggest digital systems can shorten recovery pathways and standardize tube management	High	Early postoperative risk stratification; chest‐tube strategy; earlier escalation to pleurodesis, bronchoscopy, or reoperation in selected patients	Device‐ and protocol‐dependent; thresholds are not fully standardized across centers	[35, 103, 104]
CRP	Direct PAL‐specific evidence is sparse. Available thoracic surgery data show that CRP rises after lung resection and is associated with broader postoperative outcomes, but it behaves largely as a nonspecific inflammatory marker rather than a validated PAL biomarker	Low	Adjunctive surveillance for postoperative inflammatory burden or occult complications	Poor specificity for PAL; strongly influenced by infection, surgical trauma, and comorbidity; no validated PAL threshold	[105]
IL‐6	Thoracic surgery studies show that postoperative IL‐6 can identify patients at higher risk for systemic inflammatory response and severe postoperative complications earlier than conventional markers, but PAL‐specific thresholds and prospective PAL cohorts are lacking	Low	Early postoperative signal of heightened inflammatory risk; possible future panel component	Limited PAL specificity; assay timing and availability vary; no standardized cutoff for PAL	[106, 107]
HMGB1/RAGE axis	No PAL‐specific cohorts were identified. However, mechanistic lung studies show that HMGB1 impairs epithelial barrier integrity through RAGE‐linked signaling, and experimental RAGE inhibition ameliorates acute lung injury, supporting biological plausibility for alveolar leak persistence and delayed repair	Very low	Mechanistic phenotyping; exploratory therapeutic target; hypothesis generation for epithelial‐injury pathway studies	Almost entirely translational or preclinical for PAL; no validated perioperative thresholds or sampling windows	[46, 52, 108]
sRAGE	There is no direct PAL validation, but sRAGE has relatively strong translational credibility as a marker of alveolar epithelial injury. ARDS studies and meta‐analytic data associate higher sRAGE with worse oxygenation and mortality, supporting relevance to epithelial injury phenotypes that may overlap with PAL biology	Low	Research biomarker for alveolar epithelial injury phenotyping; possible future panel component with digital drainage data	Evidence derives mainly from ARDS rather than postoperative PAL; no PAL‐specific cutoffs or prospective thoracic validation	[59, 109]
MMP‐9	Direct PAL studies are lacking. Perioperative lobectomy data show that MMP‐9 changes with surgical stress and analgesic strategy, while experimental lung‐injury work links MMP‐9 to loss of E‐cadherin and occludin, reinforcing its relevance to extracellular‐matrix remodeling and barrier disruption	Very low	Exploratory marker of remodeling and junctional injury; possible target‐enrichment variable in translational studies	Mostly indirect evidence; substantial biologic and assay heterogeneity; no PAL‐specific clinical thresholds	[9, 109]
4‐HNE/MDA	Direct PAL‐specific evidence was not identified. Experimental lung epithelial work supports 4‐HNE as a mediator of oxidative injury, and one‐lung ventilation studies indicate that perioperative oxidative stress is measurable during thoracic surgery, but translation to PAL prediction remains unproven	Very low	Exploratory oxidative‐stress phenotyping; possible inclusion in future multiparametric panels	Predominantly indirect evidence; assay variability; unclear incremental value over routine clinical data	[71, 110]
Syndecan‐1	PAL‐specific validation is absent, but lung resection studies demonstrate postoperative glycocalyx shedding, and perioperative interventions that attenuate this response may preserve barrier integrity. This makes syndecan‐1a plausible, though still early‐stage, indicator of glycocalyx and barrier injury	Low	Research marker of glycocalyx injury, capillary leak, and barrier dysfunction	Endothelial/glycocalyx signal is biologically relevant but still indirect for PAL; no validated PAL‐specific postoperative algorithm	[78, 101]
HDAC6	No clinical PAL biomarker studies were identified. Preclinical studies show that HDAC6 inhibition suppresses inflammatory signaling, limits epithelial barrier damage and remodeling, and reduces MMP‐9 upregulation, placing HDAC6 closer to an upstream therapeutic target than to a clinically deployable biomarker.	Very low	Exploratory upstream target for anti‐inflammatory and barrier‐preserving strategies	Evidence is preclinical and disease‐context dependent; no thoracic surgical validation as a biomarker	[14, 83, 102]

To further address the variability in the strength and directness of available evidence, biomarkers were additionally classified according to whether their support is derived from direct PAL‐related studies, broader perioperative evidence, indirect lung‐injury models, or preclinical mechanistic data (Table 3).

**Table 3:** Evidence‐type classification of candidate biomarkers relevant to prolonged air leak after lung resection.

Biomarker	Evidence in PAL	Evidence‐type clarification	References
Serum albumin	Direct clinical/perioperative evidence	Among the most clinically applicable markers; reflects nutritional and poor‐healing vulnerability, but remains nonspecific and may be influenced by inflammation, perioperative fluid shifts, and baseline clinical status	[3, 23]
Digital drainage parameters	Direct PAL‐specific postoperative evidence	Strongest PAL‐specific postoperative evidence; provides objective assessment of air‐leak flow, leak pattern, and pleural pressure dynamics, but cut‐offs may vary by device, protocol, and institutional drainage criteria	[29, 35]
CRP	Indirect, nonspecific perioperative inflammatory evidence	Should be interpreted primarily as a marker of systemic inflammatory burden rather than a PAL‐specific predictor; may reflect baseline disease severity, infection, or postoperative inflammation	[5]
IL‐6	Indirect perioperative morbidity/inflammatory evidence	Reflects early postoperative inflammatory activation and risk of complications, but current evidence supports its role mainly as a perioperative morbidity marker rather than a validated PAL‐specific biomarker	[5, 86]
sRAGE	Indirect evidence from ARDS/epithelial injury studies	Biologically relevant as a marker of alveolar epithelial injury, but evidence is largely derived from ARDS and acute lung injury cohorts rather than post‐resection PAL cohorts	[89]
HMGB1/RAGE	Mechanistic/preclinical and lung injury evidence	Mechanistically plausible through epithelial barrier disruption, RAGE activation, and NF‐κB‐mediated inflammation; currently hypothesis‐generating for PAL rather than clinically validated	[6, 46, 47]
MMP‐9	Translational/preclinical lung injury and ECM remodeling evidence	Supports an extracellular‐matrix‐remodeling and junction‐disruption phenotype; evidence is mainly translational, VILI‐related, or perioperative stress‐related, not directly PAL‐specific	[9, 75, 76]
Syndecan‐1	Indirect perioperative/glycocalyx injury evidence	Indicates endothelial/epithelial glycocalyx injury and barrier dysfunction; relevant to perioperative lung injury biology, but not validated as a PAL‐specific predictor	[10, 78, 79]
HDAC6	Preclinical mechanistic evidence	Least clinically ready among the listed candidates; mainly reflects upstream regulation of epithelial inflammation, barrier instability, EMT, and remodeling in preclinical or COPD‐related models	[14, 85]

*Note*: Evidence categories indicate the current directness of supporting data and should not be interpreted as validated clinical grading or definitive recommendations for routine biomarker‐guided PAL management.

Abbreviations: ARDS, acute respiratory distress syndrome; CRP, C‐reactive protein; ECM, extracellular matrix. HDAC6, histone deacetylase 6; HMGB1, high‐mobility group box 1; IL‐6, interleukin‐6; MMP‐9, matrix metalloproteinase‐9; PAL, prolonged air leak; RAGE, receptor for advanced glycation end products; sRAGE, soluble receptor for advanced glycation end products.

## 6. Pharmacological Interventions Relevant to PAL

Based on the molecular signaling and biomarkers we discussed, we are reviewing the possible pharmacological interventions to reduce the risk of PAL. It is important to mention that most of these interventions have not yet been tested clinically, and we need several studies to prove their effectiveness and safety.

### 6.1. Glycyrrhizin (GL) as HMGB1 Inhibitor

GL is an extract obtained from *Glycyrrhiza glabra* (licorice) and has been under investigation since the 1950s. Recent studies have indicated that GL possesses a range of pharmacological properties, including anti‐inflammatory, anticarcinogenic, hepatoprotective, and antiviral effects. Although the precise mechanism of action of GL remains unclear, it appears to be related to its antagonistic activity on HMGB1. GL may exhibit anti‐inflammatory and protective properties against LPS‐induced ALI in mice. GL has been shown to inhibit proinflammatory cytokines that are crucial in the early stages of the inflammatory response, indicating that the suppression of the TLR‐4/NF‐κB signaling pathway could be a potential mechanism through which GL operates. Therefore, GL presents a promising novel therapeutic approach for managing pulmonary inflammation [111]. GL significantly lowered the lung injury score, diminished I/R‐induced pulmonary permeability and isolated alveolar macrophages, and inhibited I/R‐induced inflammation in lung tissues and bronchoalveolar lavage fluid (BALF). Pre‐treatment with GL provides anti‐inflammatory and organ‐protective effects in the lung ischemia–reperfusion injury (LIRI) animal model [112]. It mitigated damage to lung tissue, decreased protein leakage, and reduced inflammation by obstructing the formation of neutrophil extracellular traps via the inhibition of the HMGB1/TLR9/MyD88 signaling pathway [113].

### 6.2. RAGE Inhibitors

RAGE antagonist peptide (RAP) improved oxidative stress, inflammation, and cell cycle arrest induced by cigarette smoke extract (CSE) in human AECs. These results indicate that the inhibition of RAGE in AECs mitigates lung injury and emphysema by reducing oxidative stress‐related inflammation and MMPs while also enhancing the proliferation of AECs [114]. In preclinical models of acid‐induced lung injury, blockade of the RAGE pathway, either by anti‐RAGE monoclonal antibodies or soluble RAGE, significantly attenuated alveolar inflammation, improved oxygenation, and restored alveolar fluid clearance (AFC) through increased aquaporin‐5 (AQP‐5) expression in type I AECs. These findings highlight the RAGE axis as a promising therapeutic target for maintaining epithelial barrier integrity and promoting alveolar healing after injury [108].

Administration of FPS‐ZM1 or azeliragon markedly suppressed the expression of RAGE and its ligands, leading to a significant reduction in LPS‐induced neutrophil‐dominant airway inflammation and tissue injury. Treatment also lowered the concentrations of proinflammatory cytokines IL‐6, IL‐1β, and TNF‐α in BALF and mitigated the elevation in alveolar–capillary permeability and pulmonary edema. Furthermore, LPS exposure profoundly disrupted airway epithelial integrity, as evidenced by the displacement of the AJ protein E‐cadherin from intercellular contacts and downregulation of both AJ and TJ proteins claudin‐2 and occludin. Remarkably, inhibition of RAGE effectively restored these structural proteins and preserved epithelial barrier continuity [51].

### 6.3. Antioxidant Therapy

Early supplementation with antioxidants, including alpha‐tocopherol and ascorbic acid, was associated with a lower incidence of organ failure and a reduced LOS in the intensive care unit among this cohort of critically ill surgical patients [115].

Following formaldehyde exposure, administration of N‐acetylcysteine (NAC) significantly reduced markers of oxidative stress, decreased the release of proinflammatory mediators, and mitigated pulmonary inflammation. Collectively, evidence from both in vitro and in vivo models demonstrates that NAC confers protective effects by modulating inflammatory responses and restoring redox balance, thereby preventing the onset of airway injury induced by formaldehyde [116]. Administration of NAC before lung resection did not reduce postoperative systemic or pulmonary inflammation nor did it mitigate oxidative damage following surgery [117]. While NAC effectively mitigated oxidative damage, reduced cellular senescence, and protected against emphysematous changes in the lung, these beneficial effects were paradoxically accompanied by a marked increase in lung adenocarcinoma incidence observed in 50% of JunD‐deficient mice and 10% of aged control mice. This duality highlights a critical caveat in antioxidant therapy; although suppression of oxidative stress can preserve tissue integrity, it may inadvertently remove reactive oxygen species‐mediated checkpoints that limit malignant transformation, particularly in genetically susceptible or aged lungs. Consequently, these findings underscore the necessity for extreme caution in translating antioxidant interventions to clinical practice, emphasizing that the long‐term oncogenic potential should be carefully weighed against short‐term protective benefits, especially in populations with chronic oxidative stress or compromised tumor suppressor pathways [118].

### 6.4. MMP‐9 Inhibitor

Various agents are known to inhibit MMP‐9, with tetracyclines representing one of the most important drug classes. Certain phytochemicals, such as quercetin and resveratrol, have also demonstrated inhibitory effects. However, in this study, we focused on doxycycline (DOX) due to the stronger and more extensive evidence supporting its efficacy [119–121].

Oral administration of DOX in mice effectively suppressed the overexpression of MMP‐9 triggered by paraquat (PQ) exposure, recapitulating the protective effects observed following neutrophil depletion. These findings suggest that DOX promotes the resolution of PQ‐induced ALI primarily by attenuating neutrophil‐derived MMP‐9 activity. Mechanistically, this highlights a critical role for MMP‐9 in mediating alveolar damage and underscores the potential of DOX as a therapeutic intervention to restore alveolar integrity in PQ‐induced lung injury. Collectively, this study provides the first experimental evidence that targeting neutrophil MMP‐9 may represent a viable strategy to mitigate ALI and potentially prevent subsequent complications such as air leak [122]. DOX preserves protective lung proteins and may block their degradation via MMP‐9 inhibition during mechanical ventilation [123]. In the study, autologous blood patch pleurodesis (ABPP) demonstrated the highest overall efficacy among the evaluated pleurodesis methods, followed by DOX, talc, and tetracycline. Both DOX and ABPP were associated with the shortest time to cessation of air leaks, while ABPP exhibited a more favorable safety profile with fewer reported complications. Overall, DOX appears to be a reliable chemical pleurodesis agent with high success rates and low recurrence; however, direct comparisons suggest that ABPP may offer superior effectiveness and safety [124].

### 6.5. Other Drug Interventions

The application of preoperative, postoperative, or life‐long administration of various drugs through different mechanisms may exhibit either beneficial or adverse effects against AL in patients following lung resection. In this section, based on the availability of data, we focus on two significant classes of drugs and discuss their efficacy in PAL.

Nonsteroidal anti‐inflammatory drugs (NSAIDs) are among the most widely prescribed medications worldwide, representing about 5% of all prescriptions. They may help reduce systemic and pulmonary inflammation, thereby lowering the risk of cellular and tissue injury as well as the incidence of PAL. However, the balance between their potential benefits and risks, along with their optimal dosage and clinical indications, requires further investigation through well‐designed clinical studies [125]. The intraoperative administration of NSAIDs was associated with a reduced incidence of postoperative pneumonia and respiratory failure, a lower rate of ICU admissions, and a shorter postoperative hospital stay [126]. Another clinical study demonstrated that NSAIDs reduce the formation of pleural adhesions. Consequently, routine intraoperative or perioperative use of NSAIDs after initial‐stage lung wedge resections may facilitate completion of second‐stage redo lobectomy by lowering the risk of dense pleural adhesions and complete pleural symphysis [127].

The one investigation showed that age, gender, and surgical technique were associated with the development of PAL. Patients with reduced FEV_1_ or DLCO, as well as those using steroids or having poor nutritional status, were less likely to experience spontaneous healing. This suggests that such patients may benefit from earlier or more proactive therapeutic intervention [21]. The use of corticosteroids showed a significant association with the occurrence of PAL, suggesting that patients receiving steroid therapy may have a higher susceptibility to developing PALs following thoracic surgery [128].

However, Zo et al. [129] showed that short‐term corticosteroid therapy was linked to reduced rates of surgical site complications, additional ICU admissions, and in‐hospital mortality. These findings highlight the importance of optimizing corticosteroid duration by carefully balancing their therapeutic benefits against potential adverse effects in the management of postoperative ALI [129].

Infectious research shows that corticosteroids exhibit dual and somewhat contradictory effects on lung tissue and epithelial barrier integrity. On one hand, corticosteroid use can induce apoptotic cell death of airway epithelial cells and increase HMGB1 levels in the airways of patients with COPD or asthma, potentially leading to epithelial barrier disruption and a higher risk of air leak following lung surgery. On the other hand, several studies have shown that glucocorticoids may enhance epithelial barrier function by strengthening TJs and increasing paracellular chloride selectivity through claudin‐8–dependent recruitment of occludin. Moreover, corticosteroid therapy has been reported to reduce MMP‐9 and increase TIMP‐1 expression in bronchial tissues, which could protect against extracellular matrix degradation and tissue damage. Therefore, the impact of corticosteroids on air leak formation or prevention appears bidirectional and dependent on factors, such as dose, duration, and patient condition, highlighting the need for further clinical research to define their optimal use [130–133]. The pharmacological strategies relevant to the prevention and treatment of PAL are outlined in Table 4.

**Table 4:** Pharmacological interventions relevant to the prevention and management of prolonged air leak after lung resection.

Pharmacological agent	Class/mechanism	Route of administration	Proposed effect on PAL	Evidence of efficacy in thoracic surgery	Timing of administration	References
Talc (magnesium silicate)	Sclerosing agent: induces pleural fibrosis via inflammation	Intrapleural (slurry via chest tube or insufflated powder)	Promotes pleurodesis: adheres lung surfaces and seals pleural air leaks	Widely used with high success rates (~60%–90%). A large 2024 study found talc pleurodesis had a lower retreatment rate than OK‐432 (talc failure ~31% vs. OK‐432–37%). Early postoperative talc pleurodesis also significantly shortened chest tube duration in patients with PAL	Postoperative (if air leak persists beyond~5–7 days).	[134, 135]
Doxycycline (tetracycline antibiotic)	Chemical pleurodesis agent; causes aseptic pleuritis and fibrosis	Intrapleural (via chest tube instillation)	Inflammation and fibrous adhesion of the pleura to seal alveolar air leaks	Recognized pleurodesis option with moderate success. A randomized trial showed that doxycycline pleurodesis shortened PAL compared with drainage‐only, though it was less effective and slower than povidone–iodine in achieving leak cessation. Still, tetracycline pleurodesis is widely reported as a standard management for persistent air leaks	Postoperative (after surgery, if a leak remains persistent, typically around day 5 of chest drainage)	[136, 137]
Povidone–iodine	Antiseptic sclerosant; provokes pleural inflammation	Intrapleural (aqueous iodine solution via chest tube)	Triggers pleural irritation and fibrosis, promoting pleural seal (chemical pleurodesis)	Demonstrated to be highly effective in trials. In an RCT for PAL, iodine pleurodesis significantly reduced air‐leak duration and hospital stay compared with observation and doxycycline. Another study reported chest tube removal ~6 days earlier with iodine vs no pleurodesis (median 9.2 vs. 15.6 days). Mild fever/chest pain are noted side effects, with no severe complications reported	Postoperative (typically used around day 5–7 if an air leak persists)	[137, 138]
OK‐432 (Picibanil; streptococcal toxin)	Immunomodulator: induces an intense inflammatory reaction in the pleura	Intrapleural (via chest tube, often reconstituted powder in saline)	Causes pleural fibrosis and adhesion, thereby sealing bronchopleural fistulas and alveolar leaks	Documented success in persistent air leak cases (historical series report ~90% leak closure). A nationwide 2024 study found that talc slightly outperformed OK‐432 in efficacy (higher success rate and fewer additional interventions needed with talc). However, other analyses indicate OK‐432 achieves comparable outcomes to talc pleurodesis in post‐resection PAL management	Postoperative (used as a chemical pleurodesis usually after a leak continues for >5 days; not typically prophylactic)	[134, 135]
*Viscum album* extract (mistletoe)	Plant‐derived cytotoxic agent; induces local inflammatory pleuritis	Intrapleural	Produces pleural irritation and fibrous adhesion formation, affecting pleurodesis to stop air leak	Proven effective in severe PAL. A prospective study in post‐lung resection PAL reported 90.4% success with single‐dose intrapleural Viscum; mean air leak cessation ~28 h and chest tubes removed ~2.8 days after pleurodesis. Side effects were limited to transient chest pain; no visceral‐related mortality (one pneumonia‐related death was deemed unrelated)	Postoperative (administered once a prolonged air leak is identified; typically, around 1 week post‐surgery if conventional management fails)	[139]
Autologous blood patch	Biological pleurodesis (patient’s own blood); fibrin clot mechanism	Intrapleural (fresh whole blood injected via chest tube, 50–120 mL)	Promotes an intrapleural fibrin clot and inflammatory reaction that seals alveolar leaks and glues pleural surfaces together	Strong evidence of efficacy. A multicenter cohort (2022) showed ABP patients had significantly shorter chest tube duration and fewer reinterventions than controls. Another trial found immediate leak cessation in ~59% of cases by the next day and shorter air leak persistence (median ~2–3 days vs. ~6 days without blood). A 2025 study reported that chest tubes were removed ~1.8 days sooner with ABP (7.8 vs. 9.5 days) and reduced hospital stay. Systematic review confirms ABP is a safe, effective PAL treatment	Postoperative (often employed early once a leak is judged “prolonged,”, e.g., on postoperative day 3–5; can be repeated every 48 h until leak stops)	[136, 140, 141]
Fresh frozen plasma (FFP)	Blood‐derived sealant; rich in clotting factors (fibrinogen) but no cells	Intrapleural (via chest tube, typically 150–200 mL FFP instilled)	FFP coagulates within the pleural space to form a fibrin patch, mechanically sealing alveolar air leaks; it also induces pleural adhesion (chemical pleurodesis effect)	Remarkable success reported in case series. A 2016 study (98 patients with post‐lobectomy PAL) saw FFP pleurodesis stop air leaks in 92% of patients within 24 h (98% by 48 h). Subsequent reports and a 2022 analysis suggest FFP is a safe, rapid alternative pleurodesis approach for persistent air leak. No significant adverse effects noted aside from transient fever/inflammation response	Postoperative (utilized as a rescue therapy once a PAL is evident, often around day 5 or earlier if other measures fail)	[136, 142]
Fibrin sealant glue (such as tisseel, autologous fibrin)	Biological adhesive; fibrinogen/thrombin‐based hemostatic sealant	Topical application on lung parenchymal suture/staple lines (sprayed or applied intraoperatively); also, via bronchoscope for internal air leaks	Creates a fibrin clot over alveolar defects, achieving immediate airtight closure and facilitating healing of the visceral pleura	Shown to reduce PAL in multiple studies. A 2024 RCT reported that adding autologous fibrin glue during lung resection significantly shortened air leak duration, chest tube days, and length of stay versus the standard technique (no sealant). Another trial comparing sealants found a very low PAL incidence in both fibrin glue groups (autologous vs. homologous); no statistical difference, confirming effective leak prevention with fibrin sealant (PAL rates <10% and no increased complications)	Intraoperative (applied prophylactically at the time of resection to prevent leaks); can also be applied bronchoscopically or via chest tube postoperatively to seal a persistent focal leak	[143, 144]
Synthetic hydrogel sealant (such as polyethylene glycol–based progel)	Polymer hydrogel sealant forms a flexible gel patch over lung tissue	Intraoperative topical application (sprayed on staple lines or air leak sites before closure)	Provides a mechanical barrier to prevent air escape from resected lung surfaces; supports tissue apposition and leak closure until healing occurs	Efficacy is mixed. In a multicenter trial, an FDA‐approved hydrogel sealed 81% of intraoperative air leaks, and 49% of patients had no postoperative leak at all. However, a retrospective study of wedge resections found no significant difference in PAL incidence or chest tube duration between patients treated with a hydrogel sealant and those without (postoperative air leak ~24% vs. 17%, *p* = 0.33). Thus, hydrogel sealants are safe and can seal intraoperative leaks, but their impact on overall PAL rates and hospital stay remains inconclusive	Intraoperative (used prophylactically at surgery if an air leak is detected or high‐risk areas are present, immediately after lung resection)	[145, 146]
Absorbable gelatin patch (TenaTac)	Bioabsorbable gelatin matrix with adhesive coating; “sealant patch”	Intraoperative application: the patch is placed over lung resection surfaces or air leak sites	Adheres to lung tissue, providing an immediate physical seal to stop air leakage; promotes coagulum formation. The patch is gradually absorbed as the tissue heals	Recent evidence indicates a major reduction in PAL with this approach. In a 2025 study, an elastic gelatin patch cut prolonged air leak incidence to 3% compared to 37% in a control group (no patch). Mean postoperative air leak duration and chest tube time were significantly shorter with the patch (air leak ~2.2 vs. 4.2 days), with no increase in complications. This suggests absorbable patches can effectively prevent most PAL when used routinely	Intraoperative (prophylactic placement at the time of resection, especially over stapler lines or fragile lung areas, before closing the chest)	[147]
50% Dextrose (glucose) solution	Hyperosmolar irritant; induces pleural inflammation via a high osmotic gradient	Intrapleural instillation (via chest tube, usually 50% glucose solution)	Causes chemical irritation and dehydration of pleural surfaces, leading to pleural inflammation and adhesion (pleurodesis) that seals air leaks	Emerging option with promising results. A 2023 trial comparing intrapleural 50% dextrose to bleomycin for PAL reported comparable efficacy in achieving leak closure, suggesting concentrated glucose can effectively induce pleurodesis. Given its low cost and availability, hypertonic glucose is noted as a potentially safe and effective alternative sclerosant for persistent air leaks (with minimal systemic absorption and adverse effects)	Postoperative (typically employed as a chemical pleurodesis after a prolonged air leak is recognized; can be used when other pleurodesis agents are contraindicated)	[136]

## 7. Toward a Biomarker‐Guided Strategy for PAL Management

Collectively, the evidence reviewed in this manuscript highlights that PAL following lung resection is not a uniform postoperative complication but rather a biologically heterogeneous process driven by variable degrees of epithelial injury, inflammatory activation, extracellular matrix remodeling, and mechanical air leak dynamics (PAL is one of the most common complications with variable incidence and multifactorial risk patterns) [136]. The persistent reliance on empiric, time‐based management strategies fails to reflect this complexity and contributes to delayed or suboptimal intervention in selected patients, as shown in systematic analyses of risk factors and prevention strategies demonstrating significant heterogeneity in outcomes and a lack of consensus on optimal management algorithms [148]. Integrating clinical risk factors with emerging biomarkers and quantitative air leak assessment, particularly using digital chest drainage systems that provide objective flow measurements and prognostic patterns, offers a pragmatic framework for biologically informed decision‐making across the perioperative continuum (quantitative air leak patterns were predictive of prolonged leakage) [31]. Such an approach enables earlier identification of patients with impaired healing capacity, supports dynamic postoperative risk re‐stratification, and facilitates phenotype‐adapted intervention once PAL becomes established. Importantly, this strategy also provides a translational foundation for future biomarker‐enriched trials of targeted pharmacologic or adjunctive therapies, while acknowledging that most candidate interventions remain investigational. Positioning biomarkers as tools for stratification rather than stand‐alone decision makers allows precision to be introduced incrementally, balancing innovation with clinical feasibility and evidentiary rigor. To translate this concept into a more practical perioperative decision‐support model, a pragmatic algorithm may be structured as follows.

**Figure 2:**
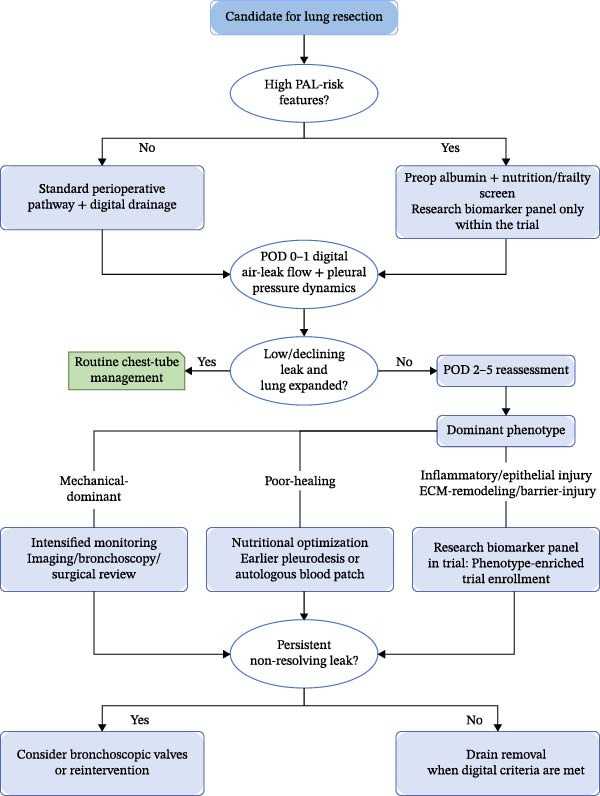
Proposed perioperative decision‐support algorithm for biomarker‐guided management of prolonged air leak after lung resection. The framework integrates preoperative risk features, serum albumin and nutritional/frailty assessment, early postoperative digital air‐leak flow and pleural pressure dynamics, and POD 2–5 reassessment to classify patients into mechanical‐dominant, poor‐healing, inflammatory/epithelial‐injury, extracellular‐matrix‐remodeling, or barrier‐injury phenotypes. The proposed algorithm is conceptual and requires prospective validation before routine clinical implementation.

A pragmatic perioperative PAL algorithm should reserve intensified biomarker assessment for patients with established or suspected healing vulnerability, including COPD/emphysema, low FEV1/DLCO or low FEV1/FVC, chronic steroid exposure, hypoalbuminemia, poor nutritional or frailty status, pleural adhesions, incomplete fissures, larger anatomic resections, and a substantial air leak on POD 0–1 digital drainage [20, 21, 23, 40, 149]. Preoperatively, serum albumin should be obtained routinely, and nutrition/frailty assessment may help identify a poor‐healing phenotype; by contrast, panels incorporating CRP, IL‐6, HMGB1/RAGE, sRAGE, MMP‐9, SDC‐1, oxidative‐stress markers, or HDAC6 should currently be restricted to biomarker‐enriched trials because their supporting evidence is largely perioperative‐inflammatory or translational rather than PAL‐specific [10, 23, 27, 102]. Immediately after resection, digital systems should quantify airflow and pleural pressure behavior during POD 0–1 because early air‐leak patterns, drainage strategy, and resolution criteria meaningfully stratify persistence and the likelihood of intervention [42, 103, 150, 151]. When integrated with clinical context and reassessed on POD 2–5, these data can frame a mechanical‐dominant phenotype, characterized by continued high flow, incomplete expansion, or worsening subcutaneous emphysema, versus poor‐healing, inflammatory/epithelial‐injury, ECM‐remodeling, or barrier‐injury phenotypes marked by persistent lower‐grade leaks in biologically vulnerable patients [1, 42, 43, 103, 150]. Mechanical‐dominant cases should prompt intensified monitoring, imaging, and bronchoscopic or surgical review, whereas poor‐healing phenotypes may justify nutritional optimization and earlier pleurodesis or autologous blood patch, with selected nonoperative candidates considered for bronchoscopic valves or trial enrollment [134, 152, 153]. This framework remains conceptual and hypothesis‐generating because validated PAL‐specific biomarker thresholds, standardized sampling windows, and biomarker‐triggered interventions are not yet available and require prospective validation. To translate this concept into a clinically oriented framework, we propose a perioperative decision‐support algorithm integrating baseline clinical risk, selected biomarker assessment, and early digital drainage parameters for PAL management (Figure 2).

## 8. Limitations and Future Directions

This narrative review has several inherent limitations. First, the available evidence on PAL remains heterogeneous, with most data derived from retrospective analyses, small prospective cohorts, or extrapolation from related pulmonary injury models rather than PAL‐specific randomized trials. Second, many of the discussed biomarkers and pharmacologic targets are supported primarily by preclinical or translational studies, limiting their immediate applicability to routine clinical practice. In addition, the lack of PAL‐specific prospective validation remains a major limitation, as several proposed biomarkers are supported mainly by indirect evidence from related lung injury models rather than direct post‐resection PAL cohorts. Moreover, clinically actionable cut‐off values, standardized sampling windows, and validated thresholds for integrating biomarker data with digital air‐leak measurements have not yet been established. Finally, the absence of standardized definitions and quantitative thresholds for air leak severity and biomarker cut‐offs constrains the generalizability of the proposed stratification frameworks. These limitations underscore the need for cautious interpretation and highlight that the concepts presented herein are hypothesis‐generating rather than prescriptive.

Future research should focus on prospective validation of biomarker‐informed risk stratification models and their integration with quantitative air leak assessment using digital drainage systems. In particular, future studies should test whether combined clinical–biomarker–digital drainage models outperform conventional clinical risk scores or isolated digital air‐leak measurements in predicting PAL persistence. Priority should also be given to defining reproducible sampling windows, especially preoperatively, on POD 0–1 and during POD 2–5, and to determining whether dynamic biomarker changes provide incremental prognostic value beyond baseline clinical risk factors and digital drainage trajectories. Biomarker‐enriched clinical trials are needed to determine whether biologically tailored interventions can meaningfully reduce PAL duration, complications, and healthcare utilization compared with those of current empiric strategies. Emphasis should be placed on identifying robust, clinically feasible biomarkers and defining actionable thresholds that enable timely, phenotype‐adapted interventions while preserving patient safety and surgical standards.

## 9. Conclusion

PAL remains a frequent and clinically consequential complication after lung resection, reflecting a complex interplay among patient‐specific biological vulnerability, parenchymal injury, inflammatory responses, and mechanical air‐leak dynamics. The evidence synthesized in this review underscores that PAL is not a uniform entity and that continued reliance on empiric, time‐based management strategies may overlook meaningful biological heterogeneity. Integrating clinical risk factors with emerging biomarkers and quantitative air‐leak assessment provides a rational framework to advance PAL management toward a more personalized, biologically informed approach. While most biomarker‐driven interventions remain investigational, their incorporation into prospective, biomarker‐enriched study designs represents a critical next step to determine whether precision‐based strategies can improve PAL‐related outcomes. Until such evidence becomes available, biomarker‐guided concepts should be viewed as complementary tools to established surgical principles, informing future research rather than replacing current standards of care.

**Nomenclature**
PAL: Prolonged air leak
APF: Alveolar–pleural fistula
VATS: Video‐assisted thoracoscopic surgery
ICU: Intensive care unit
LOS: Length of stay
COPD: Chronic obstructive pulmonary disease
FEV_1_: Forced expiratory volume in 1 s
DLCO: Diffusing capacity of the lung for carbon monoxide
CRP: C‐reactive protein
IL‐6: Interleukin‐6
TNF‐α: Tumor necrosis factor alpha
HFNC: High‐flow nasal cannula
CT: Computed tomography
HMGB1: High‐mobility group box 1 protein
DAMP: Damage‐associated molecular pattern
RAGE: Receptor for advanced glycation end products
sRAGE: Soluble receptor for advanced glycation end products
TLR: Toll‐like receptor
TLR2/TLR4/TLR9: Toll‐like receptor 2/4/9
AECs: Alveolar epithelial cells
ARDS: Acute respiratory distress syndrome
Alb: Albumin
4‐HNE: 4‐Hydroxynonenal
MDA: Malondialdehyde
ROS: Reactive oxygen species
MMP: Matrix metalloproteinase
MMP‐9: Matrix metalloproteinase 9
TIMP‐1: Tissue inhibitor of metalloproteinases 1
VILI: Ventilator‐induced lung injury
PVB: Paravertebral block
HSPG: Heparan sulfate proteoglycan
CSPG: Chondroitin sulfate proteoglycan
SDC‐1: Syndecan‐1
EGL: Endothelial glycocalyx layer
ALI: Acute lung injury
HDAC6: Histone deacetylase 6
EMT: Epithelial–mesenchymal transition
TGF‐β: Transforming growth factor beta
NF‐κB: Nuclear factor kappa‐B
JNK: c‐Jun N‐terminal kinase
GL: Glycyrrhizin
LPS: Lipopolysaccharide
BALF: Bronchoalveolar lavage fluid
LIRI: Lung ischemia–reperfusion injury
RAP: RAGE antagonist peptide
CSE: Cigarette smoke extract
AQP‐5: Aquaporin‐5
AFC: Alveolar fluid clearance
NAC: N‐acetylcysteine
NSAIDs: Nonsteroidal anti‐inflammatory drugs
ABPP/ABP: Autologous blood patch pleurodesis
FFP: Fresh frozen plasma
RCT: Randomized controlled trial.

## Author Contributions

Study concept and design: Mahdi Ahmadinia, Seyed Hootan Hamidi, Faezeh Jamali, and Hamidreza Jamaati. Data collection and analysis: Behnaz Gholizadeh Niari and Ali Bozorg Savoji. Interpretation of data: Faezeh Jamali, Hamidreza Jamaati, and Amir Farrokhian. Manuscript drafting: Mahdi Ahmadinia and Seyed Hootan Hamidi. Critical revision: Behnaz Gholizadeh Niari, Ali Bozorg Savoji, and Maryam Abbasi.

## Funding

This research did not receive any specific grant from public, commercial, or not‐for‐profit funding agencies.

## Disclosure

All authors have approved the final version of the manuscript and agree to be accountable for all aspects of the work.

## Ethics Statement

The authors have nothing to report.

## Consent

The authors have nothing to report.

## Conflicts of Interest

The authors declare no conflicts of interest.

## Data Availability

No new data were generated or analyzed in this study. Data sharing is therefore not applicable to this article. All evidence and information discussed in this narrative review were obtained from previously published studies that are cited in the manuscript.
